# Arctigenin Suppressed Epithelial-Mesenchymal Transition Through Wnt3a/β-Catenin Pathway in PQ-Induced Pulmonary Fibrosis

**DOI:** 10.3389/fphar.2020.584098

**Published:** 2020-12-16

**Authors:** Fei Gao, Yun Zhang, Zhizhou Yang, Mengmeng Wang, Zhiyi Zhou, Wei Zhang, Yi Ren, Xiaoqin Han, Mei Wei, Zhaorui Sun, Shinan Nie

**Affiliations:** ^1^ Department of Emergency Medicine, Jinling Clinical College of Nanjing Medical University, Nanjing, China; ^2^ Department of Emergency Medicine, Jinling Hospital, Medical School of Nanjing University, Nanjing, China; ^3^ Department of Emergency Medicine, Wuxi People’s Hospital Affiliated to Nanjing Medical University, Wuxi, China; ^4^ Department of Pathology, Wuxi People’s Hospital Affiliated to Nanjing Medical University, Wuxi, China

**Keywords:** paraquat, pulmonary fibrosis, arctigenin, epithelial-mesenchymal transition, Wnt3a/β-catenin pathway

## Abstract

Arctigenin (ATG), a major bioactive substance of Fructus Arctii, counters renal fibrosis; however, whether it protects against paraquat (PQ)-induced lung fibrosis remains unknown. The present study was to determine the effect of ATG on PQ-induced lung fibrosis in a mouse model and the underlying mechanism. Firstly, we found that ATG suppressed PQ-induced pulmonary fibrosis by blocking the epithelial-mesenchymal transition (EMT). ATG reduced the expressions of Vimentin and α-SMA (lung fibrosis markers) induced by PQ and restored the expressions of E-cadherin and Occludin (two epithelial markers) *in vivo* and *in vitro*. Besides, the Wnt3a/β-catenin signaling pathway was significantly activated in PQ induced pulmonary fibrosis. Further analysis showed that pretreatment of ATG profoundly abrogated PQ-induced EMT-like phenotypes and behaviors in A549 cells. The Wnt3a/β-catenin signaling pathway was repressed by ATG treatment. The overexpression of Wnt3a could weaken the therapeutic effect of ATG in A549 cells. These findings suggested that ATG could serve as a new therapeutic candidate to inhibit or even reverse EMT-like changes in alveolar type II cells during PQ-induced lung fibrosis, and unraveled that the Wnt3a/β-catenin pathway might be a mechanistic tool for ATG to control pulmonary fibrosis.

## Introduction

Paraquat (PQ) is a fast-acting and non-selective contact herbicide that has been used in more than 120 countries since the 1960s (Dinis-Oliveira RJ et al., 2008). Although its use has been banned or severely restricted, PQ self-poisoning is still frequent. It is reported the number of suicide attempts did not change after the ban in 2007 in France (Kervégant et al., 2013), and approximately 20% of the farmers continue to use PQ regardless of the ban in Korea (Bang et al., 2017). The government of China banned the sale and use of PQ in July 2016; however, there are still sporadic cases of poisoning by PQ in stock (Chen et al., 2019).

Lacking effective therapies, PQ poisoning ends up a mortality rate as high as 60%–80% (Weng et al., 2013; Zyoud, 2018). Respiratory failure is a predominant cause of PQ-induced death, because PQ, once ingested, accumulates in the lung through the polyamine uptake system (Dinis-Oliveira RJ et al., 2008). Acute lung injury (ALI) arises, characterized by severe hypoxia, edema and dysfunction at the respiratory level. Subsequently, ALI progresses to pulmonary fibrosis, causing permanent loss of lung tissue (Sun and Chen, 2016). However, the mechanism through which PQ induces pulmonary fibrosis has not been totally elucidated.

A common feature of pulmonary fibrosis is excessive proliferation of fibroblasts around the air sacs of the lungs (Barkauskas and Noble, 2014). The contribution of epithelial cells to the pool of fibroblasts and myofibroblasts in fibrotic mouse lungs remains controversial, as evidenced by some studies showing no contribution (Yamada et al., 2008; Rock et al., 2011) and others showing major contribution (Tanjore et al., 2009). Recent single cell sequencing studies showed that epithelial shape changed and the expression of SNAI1, Vimentin and planar polarity genes increased, indicating a partial EMT-like phenotype in epithelial cells of pulmonary fibrosis patients (Xu et al., 2016). These epithelial cells may activate lung fibroblasts to increase matrix deposition (Habiel et al., 2018). Previous studies also demonstrated that EMT played an important role in lung fibrosis induced by PQ (Yamada et al., 2015; Li et al., 2017b), and reversing EMT cytotype attenuated this pulmonary fibrosis (Chen et al., 2018a).

The molecular mechanisms implicated in pulmonary fibrosis and EMT have been well studied (Chanda et al., 2019). TGF-β, Wnt, hedgehog, Notch, and fibroblast growth factor (FGF) signaling pathways are associated with the development of lung fibrosis. Wnt proteins bind to Frizzled (cell surface receptors) and inactivates GSK-3βby phosphorylating GSK-3β, which further fails the phosphorylation of β-catenin. This process leads to the accumulation of hypophosphorylated β-catenin in the cytoplasm and subsequent translocation to the nucleus, where it regulates target gene expression (Hlsken and Behrens, 2000). Canonical Wnt signaling regulates a diverse set of genes, including matrix metalloproteinases (MMPs) (Wu et al., 2007), cell-cycle regulators (Davidson and Niehrs, 2010), oncogenes (Zhan et al., 2017), and angiogenic growth factors (Qu et al., 2014). Recent studies showed that Wnt signaling played key roles in regulating EMT and pulmonary fibrosis.

Arctigenin (ATG), a lignan-derived compound, is the main element of Fructus Arctii (Gao et al., 2018). Over several decades, the therapeutic effects of ATG have been extensively studied in both *in vitro* and *in vivo* models of inflammation (Tsai et al., 2011; Hyam et al., 2013), infection (Tsai et al., 2011), malignant tumor (Han et al., 2016; Xu et al., 2017), metabolic disorders (Zhong et al., 2019), and central nervous system dysfunctions (Zhu et al., 2013). Recent studies showed that ATG could reverse TGF-β1-triggered renal tubular EMT-like phenotypic changes *in vitro* (Li et al., 2015), and suppress renal interstitial fibrosis in a rat model of obstructive nephropathy (Li et al., 2017a). In addition, ATG also repressed TGF-β-induced EMT in human lung cancer cells (Xu et al., 2017). Based on these, we hypothesized that ATG could attenuate PQ-induced EMT and pulmonary fibrosis. Therefore, we designed this study to evaluate how ATG protects against PQ-induced pulmonary fibrosis.

## Materials and Methods

### Animals

Male C57BL/6 mice (201913212, Changzhou, China) without pathogens were purchased from the CAVENS Lab Animal Co. Ltd. and maintained under controlled conditions (indoor temperature of 22 ± 1°C and 50–60% humidity) and a 12-h dark/light cycle. The mice were fed with standard laboratory chow and water. The study was approved by the Institutional Animal Care and Use Committee of Wuxi People’s Hospital Afflicted to Nanjing Medical University.

### Reagents

Arctigenin was obtained from Nanjing Zelang Medical Technology Co. Ltd. (Nanjing, China). Paraquat dichloride was purchased from Sigma-Aldrich (St. Louis, MO, USA). Antibodies against the following proteins were used: Occludin, α-SMA, Wnt3a, β-Catenin, GSK-3β, P-GSK-3β, β-actin (Abcam, United States). Cytokeratin 18(Proteintech group Inc., United States), E-cadherin, Vimentin (Cell Signaling Technology, Beverly, MA, United States), Horseradish peroxidase (HRP)-conjugated goat anti-mouse and goat anti-rabbit IgG were obtained from Santa Cruz Biotechnogy (Santa Cruz, CA, United States). HYP kit (Nanjing Jiancheng Bioengineering Institute) Dulbecco’s modified Eagle’s medium (DMEM) and fetal bovine serum (FBS) were provided by Gibco (Rockville, MD, United States). All other chemicals and reagents in this study were of analytical grade.

### Induction of Pulmonary Fibrosis and Treatment Protocol

Mice were randomly divided into four groups, eight mice per group: NC group (sterile saline), PQ group (PQ 20 mg/kg), PQ+ATG low group (PQ 20 mg/kg + ATG 1 mg/kg), PQ+ATG high group (PQ 20 mg/kg + ATG 3 mg/kg). Pulmonary fibrosis was induced by single PQ administration (20 mg/kg, ip) and the analyses were performed 28 days after the PQ injection. Control animals received sterile saline at day 1. The ATG was first dissolved in dimethyl sulfoxide (DMSO) and then diluted in saline, the final volume of DMSO in saline is 2.5%, and this served as the vehicle. The mice were administered ATG or vehicle by oral gavage for 28 consecutive days started one day after PQ injection.

### Histopathological Examination

Paraffin-embedded sections of lung tissues (4 μm thickness) were deparaffinized and stained by hematoxylin and eosin (H&E) using standard methods. The sections were observed and photographed with a microscope (Olympus) at ×40 and ×200 magnification. The severity of pulmonary fibrosis in lung sections stained with Masson's trichrome stains for collagen was determined by a histopathologist blinded to the protocol design. The protein expression in these tissues was then detected by immunohistochemical staining assay. Hydrated paraffin sections were incubated in EDTA at 98°C for 20 min and then in a blocking solution (5% BSA) for 30 min. The sections were incubated at 4 °C overnight with rabbit monoclonal antibody against a-SMA (1:500), E-cadherin (1:200), Vimentin (1:500), Cytokeratin 18 (1:500), and IgG (1:200), followed by incubation for 1 h with the secondary antibody (1:200) at 37°C in the dark. Following staining with 3,3′-diaminobenzidine/H_2_O_2_ and hematoxylin, sections were cleared and mounted for observation. The collagen area and expression levels of protein were analyzed with ImageJ independently by two investigators.

### Assessment of Hydroxyproline Content in the Lung Tissue

Approximately 100 mg of lung tissue samples (stored at −80°C) were hydrolyzed with 1 mL of hydrolysate and boiled for 15 min. The HYP contents in the lung tissue were determined using an HYP kit according to the manufacturer’s instructions. The HYP content was expressed as μg/mg.

### Tissue Immunofluorescence

Hydrated paraffin sections were incubated in EDTA at 98°C for 20 min and then in a blocking solution (5% BSA) for 1 h. The sections were incubated at 4°C overnight with rabbit monoclonal antibody against a-SMA (1:150), E-cadherin (1:200), followed by incubation for 1 h with goat anti-rabbit FITC and goat anti-mouse CY3 antibody (1:200) for 50 min at 37°C. The lung sections were then incubated with DAPI for 10 min and exposed to ProLong Gold Antifade Reagent. The sections were visualized using a confocal TCS SP8 microscope (Leica Microsystems, Germany).

### Cell Culture and Treatment

Human Alveolar Epithelial Cell Line (A549 Cells) were obtained from FDCC (Shanghai, China). A549 cells were cultured in DMEM supplemented with 10% FBS. The cells were plated onto 96-well (for CCK-8 assay), 24-well (for immunostaining), and 6-well plates (for migration assay and western blot). The cells were treated with PQ and/or different concentrations of ATG as described in the figure legends.

### Cell Counting Kit-8 Assay

Cell proliferation was measured using the method described previously elsewhere (Wang et al., 2018). In brief, the cells were grown onto 96-well culture dishes with media containing different concentrations of PQ or ATG for different times. 20 μL CCK-8 solution was added into each well (containing 200 μL medium), and further cultured for 2 h at 37°C. The absorbance of each group at 450 nm was detected (n = 4) using an absorbance microplate reader. This absorbance is directly proportional to the number of living cells.

### Migration Assay

To measure cell migration, A549 cells were plated onto a 6-well plate. A wound healing assay was then performed by scratching the cell layer after PQ treatment with or without ATG for 48 h. The cell migration was observed after 24 h of scratching. Distances between the two edges of the scratch were measured and normalized to the control cells treated with medium only for calculating the relative distances.

### Immunostaining for Cells

The A549 cells were washed for three times with PBS and fixed in 4% neutral buffered formaldehyde for 10  min at room temperature. The cells were blocked in 5% bovine serum albumin (BSA) for 60 min at room temperature and then incubated with E-cadherin (1:200) or Vimentin (1:200) antibody at 4°C overnight, followed by incubation for 1 h with FITC-conjugated secondary antibody in the dark. For nuclear staining, DAPI solution was maintained for 5 min. Finally, the fluorescence signals were observed under a fluorescence microscope (Nikon 80i, Japan). For staining F-actin, cells were washed with PBS and fixed in 3.7% formaldehyde for 10 min at room temperature. After permeabilization with 0.1% Triton X-100 in PBS for 5 min, the cells were stained for 60 min with F-actin before a final wash in PBS.

### Western-Blot Analysis

Total protein from the mouse lung tissue samples and A549 cells from each group were collected and extracted using RIPA lysis buffer supplemented with PMSF (Beyotime). The protein was quantified with BCA protein assay kits with BSA as the standard. A total of 50 mg of protein from each sample were separated by 10% SDS-PAGE and transferred onto the PVDF membrane (Millipore, Bedford, MA, United States). After being blocked with 5% non-fat dried milk in TBST for 2 h at room temperature, membranes were incubated overnight at 4°C with rabbit monoclonal antibody against Vimentin (1:1,000), Occludin (1:1,000), E-cadherin (1:1,000), Wnt3a (1:1,000), β-catenin (1:5,000), GSK-3β (1:5,000), pGSK-3β (1:10,000), β-actin (1:2,000). Subsequently, membranes were incubated for 1 h at room temperature with a 1:4,000 dilution of secondary HRP-conjugated anti-rabbit or anti-mouse IgG. The immune complexes were detected using ECL reagents. All experiments were repeated for at least three times. The protein expression levels were quantified by ImageJ software.

### Construction and Transfection of Expression Plasmids

Wnt3a overexpression plasmid was constructed by inserting Wnt3a cDNA clone into a cytomegalovirus pCMV6-XL5 vector (OriGene Technologies, Rockville, MD, United States); the resulting plasmid was called pCMV6-XL5-Wnt3a. A549 cells were seeded in 6-well plates at a density of 3 × 10^5^ cells/well and transfected at approximately 50% confluence with pCMV6-XL5-Wnt3a in Opti-MEM solution (Gibco) mixed with transfection reagent MegaTran 1.0 (OriGene) at a ratio of 1:3. After incubation for 24 h, the extent of Wnt3a upregulation due to the transfection was assessed by Western blotting analysis. Cells were pretreated with DMSO or ATG and then stimulated with PQ for 48 h as described above. Cultures were then analyzed by Western blotting as described above.

### Statistical Analysis

Data were expressed as the mean ± standard deviation (SD). Using SPSS 19.0 software (Chicago, IL, United States), the data were initially subjected to one-way analysis of variance (ANOVA) for multiple comparisons, then to Dunnett’s test for selected pairs if appropriate. *p* < 0.05 was considering statistically significant.

## Results

### Arctigenin Suppressed PQ-Induced Pulmonary Fibrosis in Mice

**FIGURE 1:**
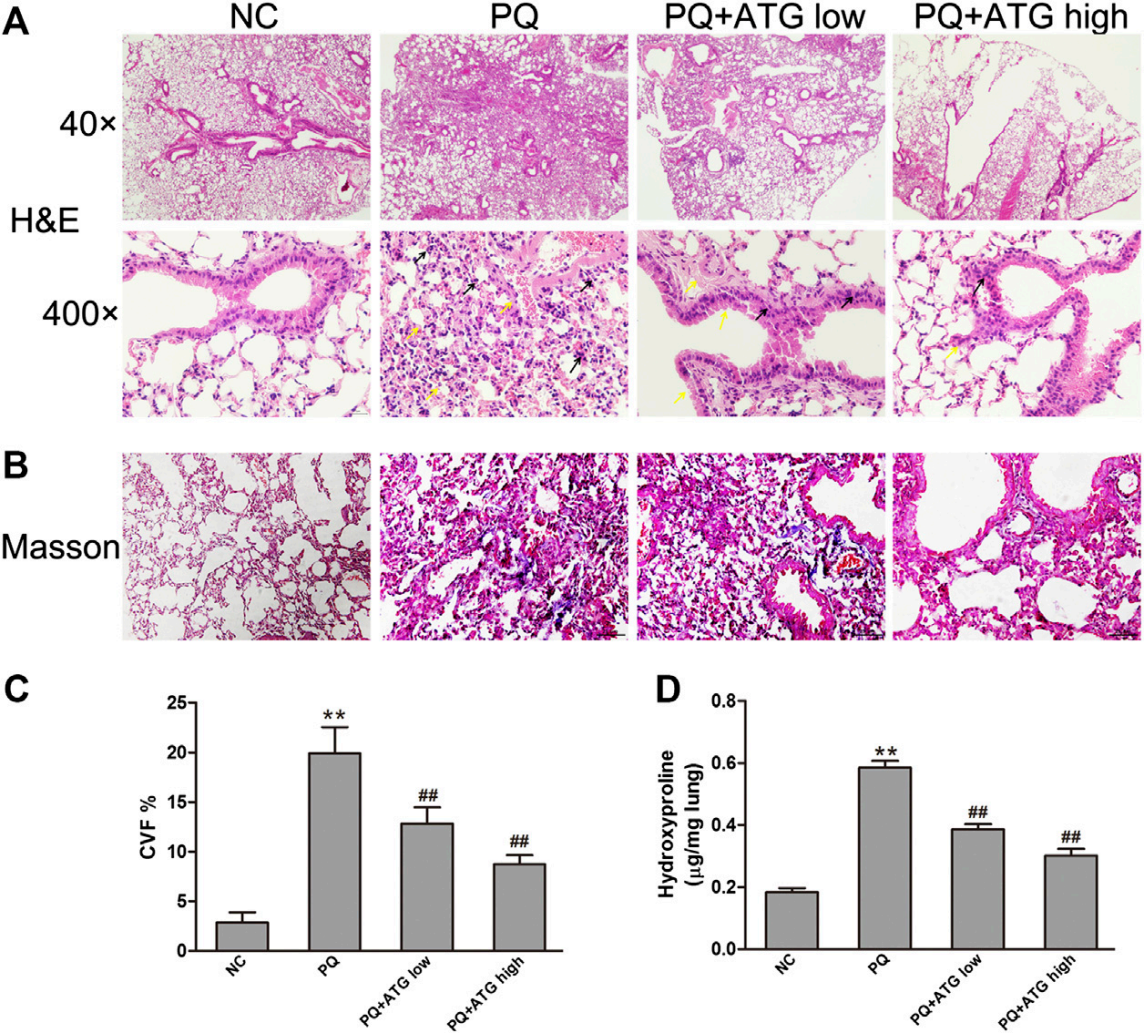
ATG suppressed PQ-induced pulmonary fibrosis in mice. Pulmonary fibrosis was induced by PQ (20 mg/kg, ip) and the analyses were performed 28 days after PQ injection. Mice in PQ + ATGlow and PQ + ATGhigh groups were administered with 1 or 3 mg/kg ATG, respectively, by oral gavage for 28 consecutive days. NC mice received sterile saline at day 1 and vehicle by oral gavage for 28 days. **(A)** H&E staining of the mice lung. Compared with NC group, the lung tissue in PQ group showed obvious interstitial inflammation, macrophages infiltration, severe interstitial fibrosis. ATG (1 and 3 mg/kg) caused noticeably alleviated pathological lung lesions produced by PQ. Black arrows showed macrophages infiltration, and yellow arrows showed interstitial fibrosis. **(B,C)** Masson’s trichrome staining of the mice lung. The collagen area and expression levels of protein were analyzed with ImageJ. **p < 0.01 versus NC group; ^##^ <0.01 versus PQ-treated group. **(D)**Assessment of hydroxyproline (HYP) content in lung tissue. **p < 0.01 versus NC group; <0.01 versus PQ-treated group.

To investigate whether ATG can suppress pulmonary fibrosis *in vivo*, histological changes in the lung were examined using H&E staining (Figure 1A). Twenty-eight days after the administration of PQ, the lung tissues showed obvious interstitial inflammation, macrophages infiltration, severe interstitial fibrosis. Treatment with ATG (1 and 3 mg/kg) caused noticeable alleviation in pathological lung lesions. The sections of the control group showed structural integrity, without evidence of inflammation or fibrosis. Masson’s trichrome stains showed PQ induced lung fibrosis, and ATG treatment significantly decreased the fibrosis (Figures 1B, C). To validate the effect of ATG, we evaluated lung fibrosis by measuring the HYP content in lungs as an index of collagen accumulation. As expected, the ATG low and ATG high groups significantly inhibited the increased HYP levels compared with the PQ group (Figure 1D).

### Arctigenin Decreased PQ-Induced Epithelial-Mesenchymal Transition in the Lungs of Mice Model

**FIGURE 2:**
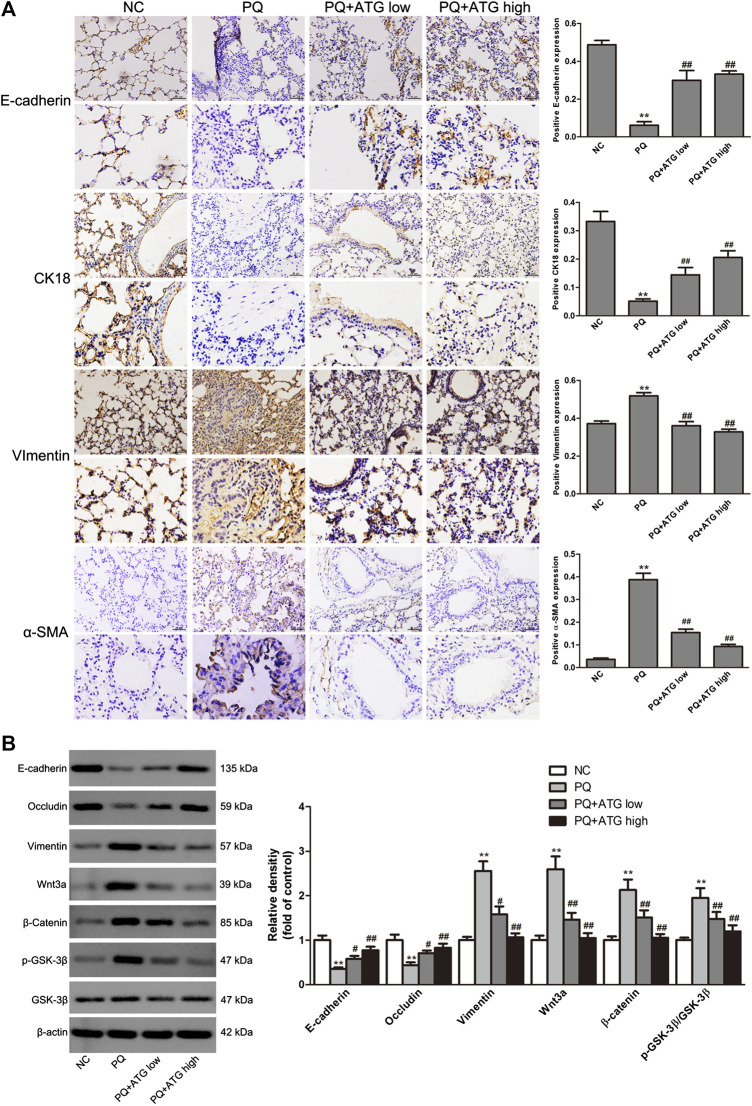
ATG decreased PQ-induced EMT in mouse lung. **(A)** The locations and expressions of E-cadherin, Cytokeratin 18, α-SMA and Vimentin were determined by immunohistochemical staining in the lung sections. **p < 0.01 versus NC group; <0.01 versus PQ-treated group. **(B)** The expressions of Vimentin, E-cadherin, Occludin, Wnt3a, β-Catenin, GSK-3β and pGSK-3β in the lung tissue of mice were evaluated by Western blot. β-actin served as an internal control. Data were presented as mean ± SD of three independent experiments. **p < 0.01 versus NC group; *p* < 0.01 versus PQ-treated group.

**FIGURE 3:**
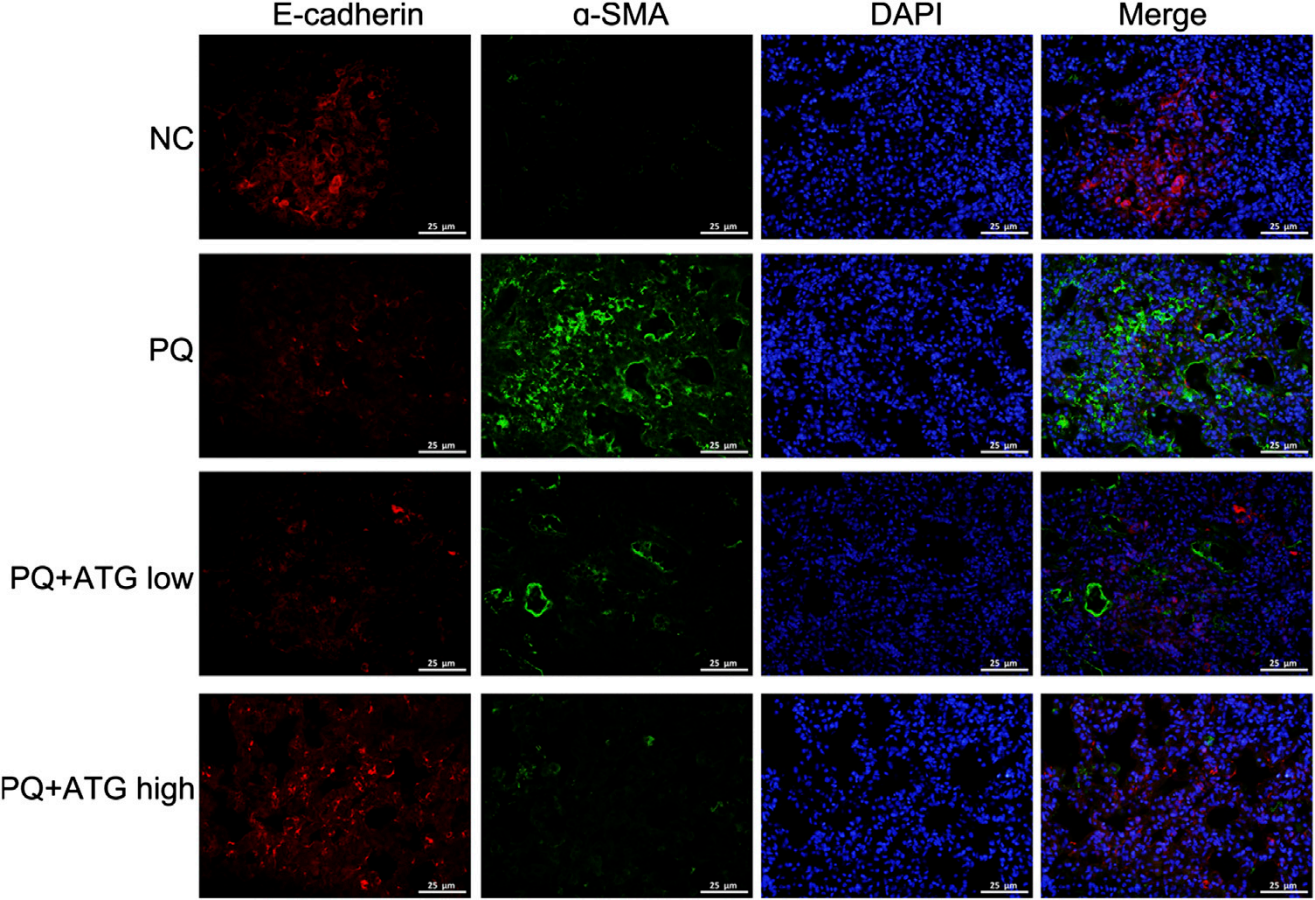
Tissue immunofluorescence. The expressions of E-cadherin and α-SMA in mice lung were analyzed by immunofluorescence.

To investigate whether ATG improved the lung fibrosis by restraining EMT, we labeled Vimentin, α-SMA, E-cadherin and Cytokeratin 18 in paraffin sections of lung tissue by immunohistochemical staining. The results showed that the expressions of Vimentin and α-SMA in PQ group were significantly increased; meanwhile, the expressions of E-cadherin and Cytokeratin 18 were reduced. Both changes were significantly reversed by ATG treatment (Figure 2A). To confirm our results, the expression levels of the Vimentin, E-cadherin and Occludin in the lung tissues of mice were evaluated by Western blot. The results showed that compared to NC group, the protein levels of E-cadherin and Occludin were decreased, while that of Vimentin was significantly increased in PQ group. Notably, these changes were reversed in ATG treatment groups, which indicated that ATG decreased PQ-induced EMT in mice (Figure 2B). Since previous studies showed that PQ poisoning promotes EMT by activating Wnt signal pathway, we hypothesized that ATG regulated PQ-induced EMT depending on Wnt3a. Subsequently, we examined the protein levels of Wnt3a, β-Catenin, GSK-3β and pGSK-3β in lung tissues by Western blot. The results indicated that the protein levels of Wnt3a, β-Catenin and pGSK-3β were increased significantly in PQ group, compared to those in the control group. ATG decreased the expressions of Wnt3a, β-Catenin and pGSK-3β in the mice model, indicating that ATG could inhibit the activation of Wnt signaling induced by PQ (Figure 2B). Tissue immunofluorescence was performed to validate the effect of ATG. As shown in Figure 3, the expression of α-SMA in PQ group was significantly increased, while the expression of E-cadherin was reduced. Both changes were significantly reversed by ATG treatment.

### Arctigenin Reversed the Progression of PQ-Induced Epithelial-Mesenchymal Transition and Down-Regulated Wnt3a Signaling Pathway in Alveolar Epithelial Cells

**FIGURE 4:**
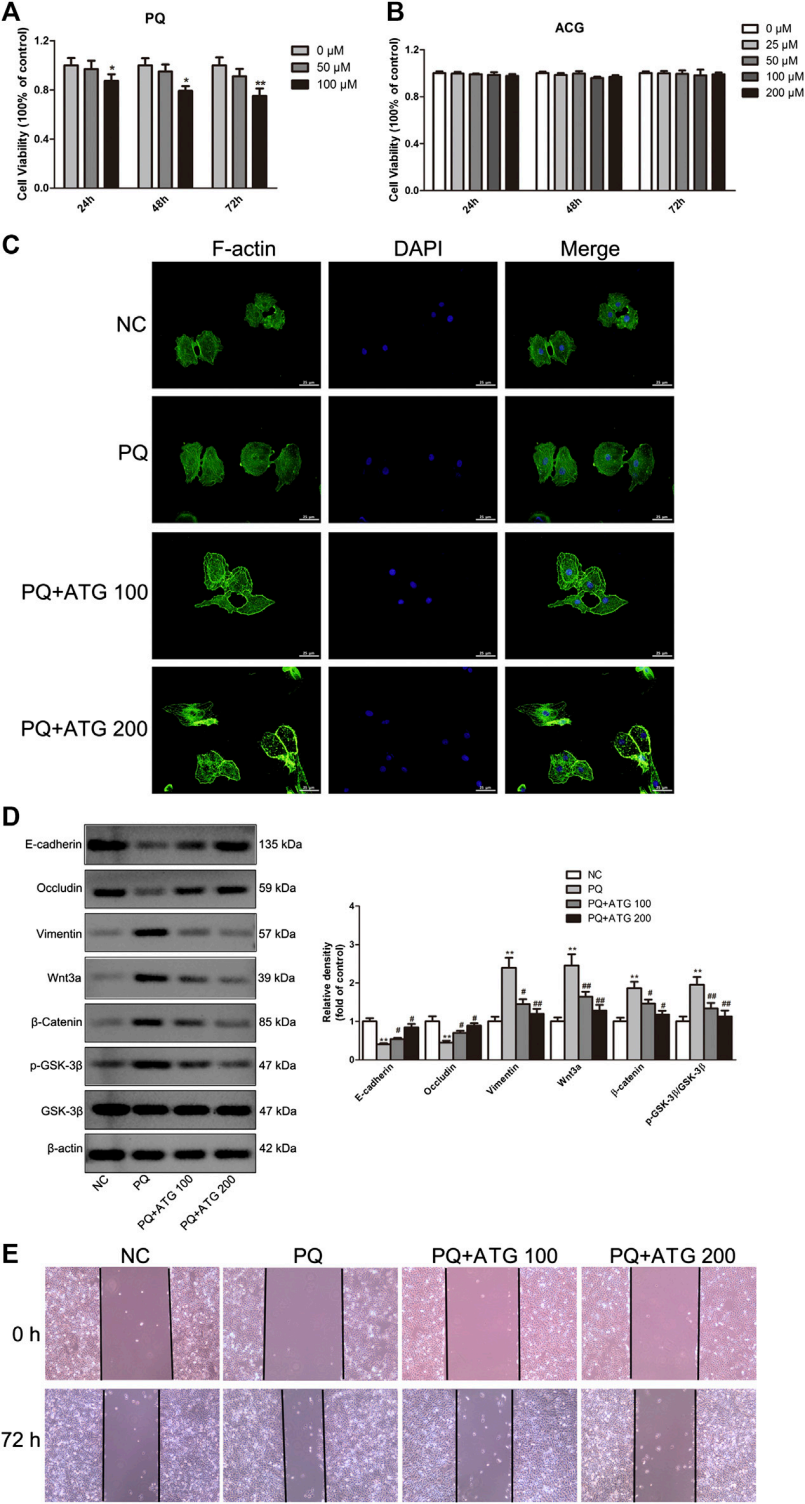
ATG reversed PQ-induced EMT in A549 cells. **(A)** Cell counting kit-8 (CCK8) was applied to measure the cytotoxicity of PQ on A549 cells at concentration of 0, 50, 100 μM for 24, 48, and 72 h, respectively. Values were represented as the means ± SD, *p < 0.05, versus 0 μM, n = 4. **(B)** Effect of ATG on cell proliferation was determined using CCK8 assay. A459 cells were treated with ATG at concentrations of 0, 25, 50, and 100 μM for 24, 48, and 72 h, respectively. Values were represented as the means ± SD, n = 4. **(C)** Phalloidin immunofluorescence was performed to show the effect of ATG on EMT in-vitro. **(D)** A549 cells were pre-treated with ATG (100 and 200 μM). Two hours later, the cells were co-incubated with 50 μM PQ for 48 h. The expression of Vimentin, E-cadherin, Occludin, Wnt3a, β-Catenin, GSK-3β and P-GSK-3β in A549 cells were evaluated by Western blot. β-actin served as an internal control. Data were presented as mean ± SD of three independent experiments. **p < 0.01 versus NC group; *p* < 0.01 versus PQ-treated control group. **(E)** After A549 cells were treated with PQ with or without different doses of ATG for 72 h, confluent epithelial monolayers were wounded and incubated with serum-free DMSO. At 24 h after wounding, cell migration was assessed using a microscope equipped with a camera.

Cell counting kit-8 (CCK8) was applied to measure the cytotoxicity of PQ on A549 cells at concentration of 0, 50, 100 μM for 24, 48 and 72 h, respectively. The results showed 100  μM PQ could inhibit the proliferation of A549 cells (Figure 4A). Thus, to confirm the suppressive role of ATG during EMT, A549 cells were treated with PQ (50 μM) to induce EMT *in vitro*. To determine the tolerated dose of ATG on A549 cells, the CCK-8 assay was conducted to detect the maximum toxicity of ATG in the A549 cell line. The results indicated that the maximal concentration of ATG (200 μM) did not cause any cytotoxicity in A549 cells (Figure 4B). We selected two doses (100 and 200 μM) of ATG for further experiments. A549 cells were pre-treated with ATG. Two hours later, the cells were co-incubated with 50 μM PQ for 72 h. To demonstrate PQ-induced EMT phenotype and the effects of ATG on EMT *in vitro*, F-actin was stained by immunostaining. As shown in Figure 4C, the cytoskeleton changed after PQ treatment, while pretreatment of ATG noticeably reversed the EMT cytotype. The normal cytoskeleton is comparatively small with many bulges on the edge. After PQ administration, the cytoskeleton became larger and the edge turned smooth. The cytoskeleton shrank and well-defined bulges appeared on the edge after low-concentration ATG was given. While after high-concentration ATG treatment, the size of the cytoskeleton was like that of NC, and multiple bulges appeared on the edge. EMT markers were further detected by Western Blot. We observed a prominent decrease in E-cadherin and Occludin expression, and a significant increase in Vimentin expression in PQ-treated A549 cells. However, pretreatment of ATG noticeably reversed the EMT cytotype in a dose-dependent manner (Figure 4D). The protein levels of Wnt3a, β-Catenin, GSK-3β and pGSK-3β in A549 cells were also investigated by Western blot, the results of which were in consistent with in-vivo experiment. Expressions of Wnt3a, β-Catenin and pGSK-3β were increased prominently in PQ-treated cells. However, ATG inhibited Wnt3a, β-Catenin and pGSK-3β expressions (Figure 4D). Since cell migration and invasion increased dramatically following EMT, we examined the effects of PQ, with and without ATG pre-treatment, on the behavior of A549 cells with wound healing assays. The widths of wounds were measured at the beginning of the assay and 24 h after wounding. The results showed that the mobility of A549 cells had increased significantly in PQ group, and then reduce by ATG (Figure 4E).

### Wnt3a Overexpression Rescued the Inhibitory Effect of Arctigenin on PQ-Induced EMT-like Phenotypic Changes in A549 Cells

**FIGURE 5:**
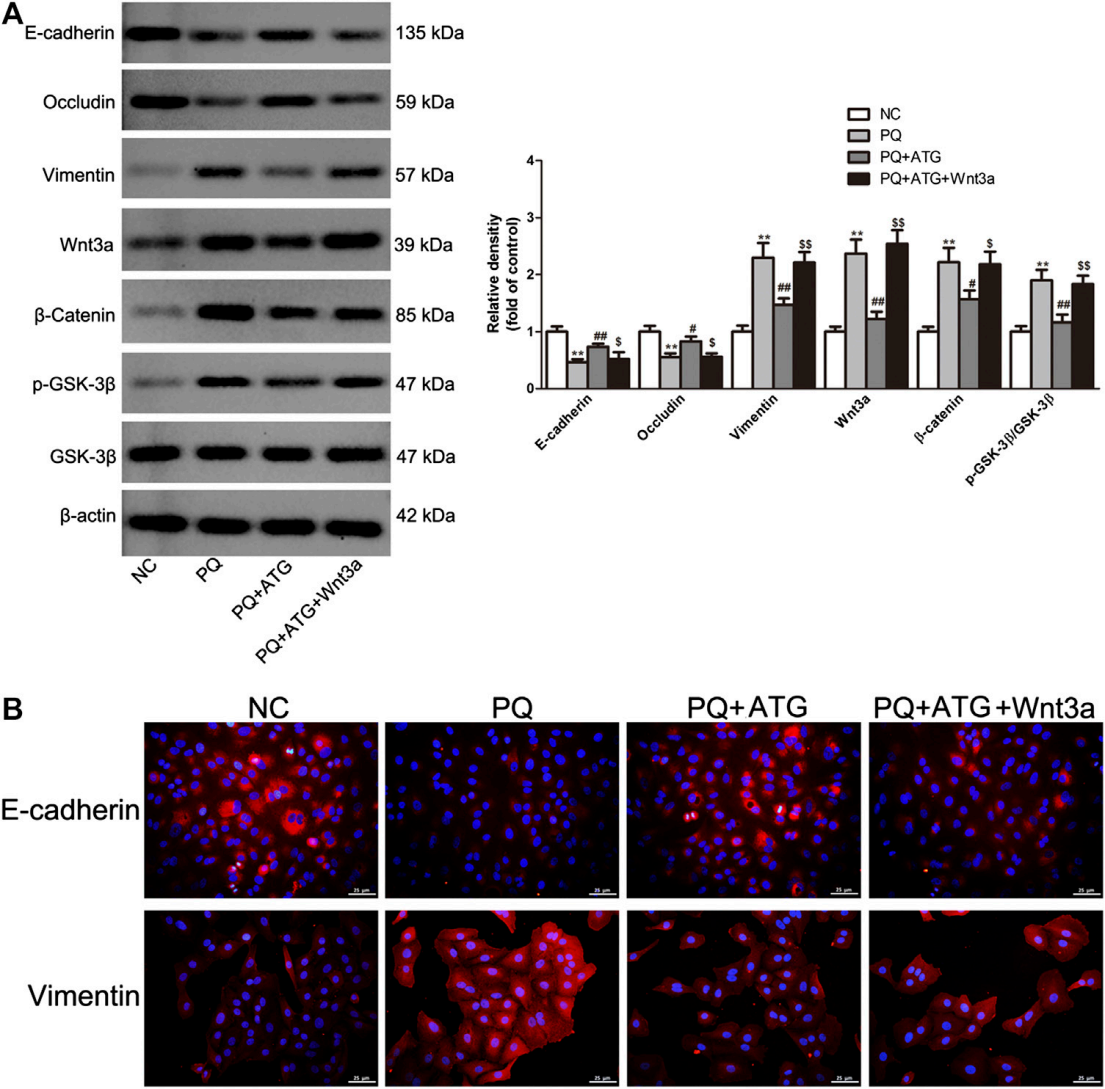
Wnt3a overexpression rescued the inhibitory effect of ATG on PQ-induced EMT-like phenotypic changes in A549 cells. **(A)** After transfection of Wnt3a overexpression plasmids for 24 h, the cells were treated in the presence or absence of PQ (50 μM) or ATG (100 μM) for 48 h. After treatment, whole cell lysates were immunoblotted with antibodies against vimentin, E-cadherin, Occludin, Wnt3a, β-Catenin, GSK-3β, P-GSK-3β and β -actin. The experiments were repeated for three times with similar results, and a representative immunoblot was shown for each protein. **p < 0.01 versus NC group; *p < 0.05 versus PQ-treated group, *p* < 0.01 versus PQ-treated group, $ *p* < 0.05 versus PQ+ATG group, $$ *p* < 0.01 versus PQ+ATG group. **(B)** The expression of E-cadherin and Vimentin were further analyzed by immunofluorescence.

The experiments described above suggest that ATG inhibits PQ-induced activation of Wnt3a/β-catenin signaling pathway, as well as EMT-like phenotypic changes. To provide more direct evidence, we transfected A549 cells with Wnt3a overexpression plasmids and then treat them with ATG and PQ. We measured expression levels of Wnt3a, β-Catenin, GSK-3β, pGSK-3β and the EMT-like phenotype markers E-cadherin, Occludin and Vimentin by Western blot. After transfected with Wnt3a overexpression plasmids, the expression levels of Wnt3a, β-Catenin, pGSK-3β, E-cadherin, Occludin and Vimentin in cells pretreated with ATG were almost restored to those in cells treated only with PQ (Figure 5A). The expressions of E-cadherin and Vimentin were further analyzed by immunofluorescence (Figure 5B). The results confirmed the vital role of Wnt3a/β-catenin signaling pathway in pulmonary fibrogenesis induced by PQ.

**FIGURE 6:**
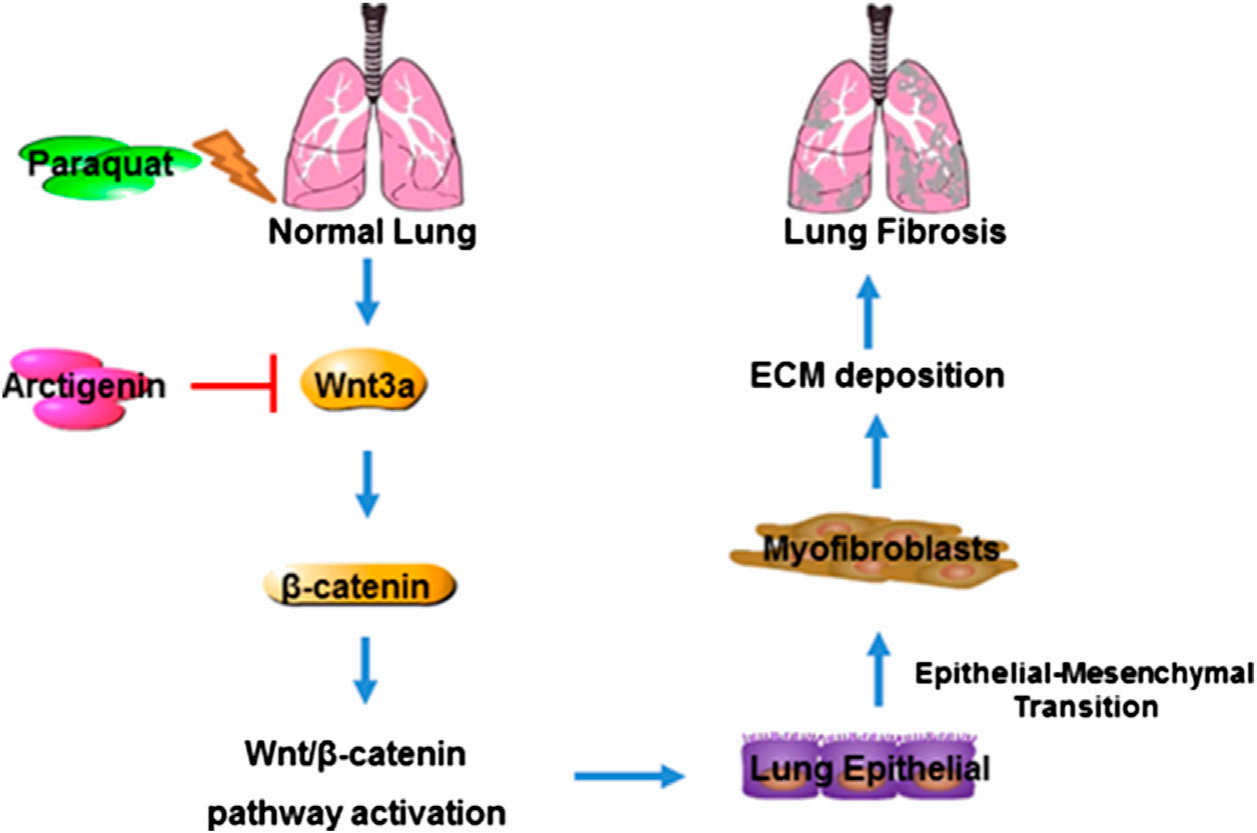
Mechanism of ATG in alleviating PQ-induced lung fibrosis. PQ induces lung fibrosis by promoting lung epithelial cells to differentiate into myofibroblasts and ECM deposition by activating Wnt3a/β-catenin signaling, whereas ATG exerts its anti-fibrotic effect through the inhibition of Wnt3a/β-catenin signaling pathway.

## Discussion

Despite its high toxicity, PQ has been commonly used as herbicides, sometimes accidentally or intentionally ingested by humans. The high mortality of PQ poisoning is attributed to the lack of effective treatment. Pulmonary fibrosis causes lung tissue loss and respiratory failure, which is the main pathology after PQ poisoning. In the present study, we found that PQ could induce pulmonary fibrosis and EMT through activating Wnt signal pathway, and ATG, as an active substance of Fructus Arctii, could attenuate PQ-induced EMT both *in vivo* and *in vitro*. The study was the first one to investigate the effect of ATG on PQ-induced pulmonary fibrosis.

EMT is a biological process during which epithelial cells lose their epithelial cell characteristics and gain mesenchymal characteristics. EMT is involved in many different pathologic and physiologic processes, including embryogenesis, tissue repair and carcinoma (Gurzu et al., 2019). During the EMT, the expression of the epithelial marker E-cadherin is silenced, while the expression of mesenchymal markers, including vimentin and matrix metalloproteinase, is increased, leading to disappeared cell-cell contacts, increased cell migratory behavior and excessive extracellular matrix production. Previous studies showed EMT played an important role in the pathogenesis of pulmonary fibrosis (Han et al., 2015; Yamada et al., 2015; Jolly et al., 2018). In this study, we investigated the EMT in PQ-induced lung fibrosis, and whether ATG could attenuate pulmonary fibrosis by alleviating EMT progress. The HE staining and Masson staining studies confirmed that PQ administration induced lung fibrosis in male C57BL/6 mice, which was then significantly alleviated by ATG. The *in vivo* study showed the expression of epithelial marker E-cadherin, Occludin and CK18 was significantly decreased, while that of Vimentin and α-SMA, as a mesenchymal marker, was significantly increased in the lung tissues of PQ-treated mice. The *in vitro* study showed a decrease in E-cadherin and Occludin and an increase in Vimentin in PQ-treated A549 cells. While ATG reversed EMT in a dose-dependent manner both *in vivo* and *in vitro*.

Wnt/β-catenin signaling has been demonstrated to function in pulmonary fibrosis. Previous studies showed Wnt signaling playing key roles in epithelial cell proliferation, EMT, myofibroblast differentiation, and collagen synthesis (Burgy and Königshoff, 2018; Chen et al., 2018b). In the present study, we found that the expressions of Wnt3a, β-catenin, WISP1 were increased in the mouse model of PQ-induced pulmonary fibrosis, and ATG administration suppressed the activation of Wnt signaling. Furthermore, an *in vitro* experiment was performed to clarify whether Wnt/β-catenin was involved in PQ-induced EMT in A549 cells. The results revealed that Wnt/β-catenin signaling was activated and the EMT was induced in PQ-treated A549 cells, and ATG treatment reversed the activation of Wnt/β-catenin signaling *in vitro* as well. The results demonstrated that Wnt/β-catenin signaling was activated in PQ-induce EMT, and inhibited by ATG treatment.

In Wnt family, 19 types of glycoproteins regulate mammalian embryonic development and regenerative responses to injury in postnatal life (Nusse and Clevers, 2017). Wnt3a represents one of the classical “canonical” Wnt ligands that trigger β-catenin signaling. It has been showed that Wnt3a was significantly upregulated in experimental and human idiopathic pulmonary fibrosis (Aumiller et al., 2013). In the present study, both *in vivo* and *in vitro* studies showed Wnt3a was significantly upregulated in PQ-induced EMT, and then suppressed by ATG. The *in vitro* study showed Wnt3a discounted the effect of ATG on EMT in Alveolar Epithelial Cell. The result suggested that ATG attenuated PQ-induced EMT by blocking the activation of Wnt3a/β-catenin signaling pathway.

Nevertheless, the present study has several limitations. Firstly, we did not examine the effects of Wnt3a overexpression in mice. In addition, we did not determine the most effective dose and safe dose range of ATG. Previous studies showed the least observed adverse effect was induced by 12 mg/kg daily ATG exposure for successive 28 days in rats (Tan et al., 2018). In the present study, no mortality and obvious clinical signs of ATG-related toxicity were observed in animals treated with ATG (3 mg/kg/d) alone during the study period (28 days, data shown in Supplementary Figure S1), suggesting that ATG may be safe to treat PQ-induced fibrosis.

In conclusion, ATG treatment could attenuate PQ-induced pulmonary fibrosis in male C57BL/6 mice. Meanwhile, ATG could block PQ-induced EMT both *in vivo* and *in vitro*, likely via regulating Wnt3a/β-catenin signaling pathway. These findings might guide to explore the therapeutic potential of ATG in pulmonary fibrosis after PQ poisoning.

## Data Availability Statement

The original contributions presented in the study are included in the article/Supplementary Material, further inquiries can be directed to the corresponding authors.

## Author Contributions

FG, YZ and ZS carried out most of the experiments; ZY and MW provided the statistical support, ZZ provided pathology technique support, WZ and YR contributed to some experiments. XH and MW contributed to the manuscript revision and data curation. SN and ZS designed the study and analyzed the data.

## Funding

This study was supported by Social Development Projects of Jiangsu Province (BE2017720), the Science Foundation of Jiangsu Health Commission (H2018039), the National Natural Science Foundation of China (81401583 and 81701894), Jiangsu Provincial Medical Youth Talent (QNRC2016909 and QNRC2016908), Natural Science Foundation of Jiangsu Province (BK20190247)，China Postdoctoral Science Foundation (2018M643890), Jiangsu Postdoctoral Science Foundation (2018K048A) and the Science Research and Technology Development General Program of Wuxi Health Commission, China (MS201714).

## Conflict of Interest

The authors declare that the research was conducted in the absence of any commercial or financial relationships that could be construed as a potential conflict of interest.

## References

[B1] Aumiller V. Balsara N. Wilhelm J. Günther A. Königshoff M. (2013). WNT/β-catenin signaling induces IL-1β expression by alveolar epithelial cells in pulmonary fibrosis. Am. J. Respir. Cell Mol. Biol. 49, 96–104. 10.1165/rcmb.2012-0524OC 23526221

[B2] Bang Y. J. Kim J. Lee W. J. (2017). Paraquat use among farmers in Korea after the ban. Arch. Environ. Occup. Health 72, 231–234. 10.1080/19338244.2016.1192982 27219666

[B3] Barkauskas C. E. Noble P. W. (2014). Cellular mechanisms of tissue fibrosis. 7. New insights into the cellular mechanisms of pulmonary fibrosis. Am. J. Physiol. Cell Physiol. 306, C987–C996. 10.1152/ajpcell.00321.2013 24740535 PMC4422352

[B4] Burgy O. Königshoff M. (2018). The WNT signaling pathways in wound healing and fibrosis. Matrix Biol. 68–69, 67–80. 10.1016/j.matbio.2018.03.017 29572156

[B5] Chanda D. Otoupalova E. Smith S. R. Volckaert T. De Langhe S. P. Thannickal V. J. (2019). Developmental pathways in the pathogenesis of lung fibrosis. Mol. Aspect. Med. 65, 56–69. 10.1016/j.mam.2018.08.004 PMC6374163 30130563

[B6] Chen F. Ye Y. Jin B. Yi B. Wei Q. Liao L. (2019). Homicidal paraquat poisoning. J. Forensic Sci. 64, 941–945. 10.1111/1556-4029.13945 30452771

[B7] Chen H. Chen Q. Jiang C. M. Shi G. Y. Sui B. W. Zhang W. (2018a). Triptolide suppresses paraquat induced idiopathic pulmonary fibrosis by inhibiting TGFB1-dependent epithelial mesenchymal transition. Toxicol. Lett. 284, 1–9. 10.1016/j.toxlet.2017.11.030 29195901

[B8] Chen X. Shi C. Cao H. Chen L. Hou J. Xiang Z. (2018b). The hedgehog and Wnt/β-catenin system machinery mediate myofibroblast differentiation of LR-MSCs in pulmonary fibrogenesis. Cell Death Dis. 9, 639 10.1038/s41419-018-0692-9 29844390 PMC5974360

[B9] Davidson G. Niehrs C. (2010). Emerging links between CDK cell cycle regulators and Wnt signaling. Trends Cell Biol. 20, 453–460. 10.1016/j.tcb.2010.05.002 20627573

[B10] Dinis-Oliveira RJ D. J. A. Sánchez-Navarro A. R. F. Bastos ML C. F. (2008). Paraquat poisonings: mechanisms of lung toxicity, clinical features, and treatment. Crit. Rev. Toxicol. 38, 13–71. 10.1080/10408440701669959 18161502

[B11] Gao Q. Yang M. Zuo Z. (2018). Overview of the anti-inflammatory effects, pharmacokinetic properties and clinical efficacies of arctigenin and arctiin from Arctium lappa L. Acta Pharmacol. Sin. 39, 787–801. 10.1038/aps.2018.32 29698388 PMC5943914

[B12] Gurzu S. Kobori L. Fodor D. Jung I. (2019). Epithelial mesenchymal and endothelial mesenchymal transitions in hepatocellular carcinoma: a review. BioMed Res. Int. 2019, 2962580 10.1155/2019/2962580 31781608 PMC6855070

[B13] Habiel D. M. Espindola M. S. Jones I. C. Coelho A. L. Stripp B. Hogaboam C. M. (2018). CCR10+ epithelial cells from idiopathic pulmonary fibrosis lungs drive remodeling. JCI Insight 3 10.1172/jci.insight.122211 PMC6141169 30135312

[B14] Han Y. H. Kee J. Y. Kim D. S. Mun J. G. Jeong M. Y. Park S. H. (2016). Arctigenin inhibits lung metastasis of colorectal cancer by regulating cell viability and metastatic phenotypes. Molecules 21 10.3390/molecules21091135 PMC6272973 27618887

[B15] Han Y. Y. Shen P. Chang W. X. (2015). Involvement of epithelial-to-mesenchymal transition and associated transforming growth factor-β/Smad signaling in paraquat-induced pulmonary fibrosis. Mol. Med. Rep. 12, 7979–7984. 10.3892/mmr.2015.4454 26499763 PMC4758328

[B16] Hlsken J. Behrens J. (2000). The Wnt signalling pathway. J. Cell Sci. 113 (Pt 20), 3545. 11017867 10.1242/jcs.113.20.3545

[B17] Hyam S. R. Lee I. A. Gu W. Kim K. A. Jeong J. J. Jang S. E. (2013). Arctigenin ameliorates inflammation *in vitro* and *in vivo* by inhibiting the PI3K/AKT pathway and polarizing M1 macrophages to M2-like macrophages. Eur. J. Pharmacol. 708, 21–29. 10.1016/j.ejphar.2013.01.014 23375938

[B18] Jolly M. K. Ward C. Eapen M. S. Myers S. Hallgren O. Levine H. (2018). Epithelial-mesenchymal transition, a spectrum of states: role in lung development, homeostasis, and disease. Dev. Dynam. 247, 346–358. 10.1002/dvdy.24541 28646553

[B19] Kervégant M. Merigot L. Glaizal M. Schmitt C. Tichadou L. de Haro L. (2013). Paraquat poisonings in France during the European ban: experience of the Poison control center in marseille. J. Med. Toxicol. 9, 144–147. 10.1007/s13181-012-0283-6 23435962 PMC3657034

[B20] Li A. Wang J. Zhu D. Zhang X. Pan R. Wang R. (2015). Arctigenin suppresses transforming growth factor-β1-induced expression of monocyte chemoattractant protein-1 and the subsequent epithelial-mesenchymal transition through reactive oxygen species-dependent ERK/NF-κB signaling pathway in renal tubular epithelial cells. Free Radic. Res. 49, 1095–1113. 10.3109/10715762.2015.1038258 25968940

[B21] Li A. Zhang X. Shu M. Wu M. Wang J. Zhang J. (2017a). Arctigenin suppresses renal interstitial fibrosis in a rat model of obstructive nephropathy. Phytomedicine 30, 28–41. 10.1016/j.phymed.2017.03.003 28545667

[B22] Li T. Yang X. Xin S. Cao Y. Wang N. (2017b). Paraquat poisoning induced pulmonary epithelial mesenchymal transition through Notch1 pathway. Sci. Rep. 7, 924 10.1038/s41598-017-01069-9 28424456 PMC5430447

[B23] Nusse R. Clevers H. (2017). Wnt/β-Catenin signaling, disease, and emerging therapeutic modalities. Cell 169, 985–999. 10.1016/j.cell.2017.05.016 28575679

[B24] Qu B. Liu B. R. DU Y. J. Chen J. Cheng Y. Q. Xu W. (2014). Wnt/β-catenin signaling pathway may regulate the expression of angiogenic growth factors in hepatocellular carcinoma. Oncol Lett 7, 1175–1178. 10.3892/ol.2014.1828 24944688 PMC3961220

[B25] Rock J. R. Barkauskas C. E. Cronce M. J. Xue Y. Harris J. R. Liang J. (2011). Multiple stromal populations contribute to pulmonary fibrosis without evidence for epithelial to mesenchymal transition. Proc. Natl. Acad. Sci. U.S.A. 108, E1475–E1483. 10.1073/pnas.1117988108 22123957 PMC3248478

[B26] Sun B. Chen Y. G. (2016). Advances in the mechanism of paraquat-induced pulmonary injury. Eur. Rev. Med. Pharmacol. Sci. 20, 1597–1602. 27160134

[B27] Tan Y. J. Ren Y. S. Gao L. Li L. F. Cui L. J. Li B. (2018). 28-Day oral chronic toxicity study of arctigenin in rats. Front. Pharmacol. 9, 1077 10.3389/fphar.2018.01077 30319414 PMC6169246

[B28] Tanjore H. Xu X. C. Polosukhin V. V. Degryse A. L. Li B. Han W. (2009). Contribution of epithelial-derived fibroblasts to bleomycin-induced lung fibrosis. Am. J. Respir. Crit. Care Med. 180, 657–665. 10.1164/rccm.200903-0322OC 19556518 PMC2753790

[B29] Tsai W. J. Chang C. T. Wang G. J. Lee T. H. Chang S. F. Lu S. C. (2011). Arctigenin from Arctium lappa inhibits interleukin-2 and interferon gene expression in primary human T lymphocytes. Chin. Med. 6, 12 10.1186/1749-8546-6-12 21435270 PMC3076299

[B30] Wang Q. Zheng M. Yin Y. Zhang W. (2018). Ghrelin stimulates hepatocyte proliferation via regulating cell cycle through gsk3β/Β-catenin signaling pathway. Cell. Physiol. Biochem. 50, 1698–1710. 10.1159/000494789 30384380

[B31] Weng C. H. Hu C. C. Lin J. L. Lin-Tan D. T. Hsu C. W. Yen T. H. (2013). Predictors of acute respiratory distress syndrome in patients with paraquat intoxication. PLoS One 8, e82695 10.1371/journal.pone.0082695 24349340 PMC3859634

[B32] Wu B. Crampton S. P. Hughes C. C. (2007). Wnt signaling induces matrix metalloproteinase expression and regulates T cell transmigration. Immunity 26, 227–239. 10.1016/j.immuni.2006.12.007 17306568 PMC1855210

[B33] Xu Y. Lou Z. Lee S. H. (2017). Arctigenin represses TGF-β-induced epithelial mesenchymal transition in human lung cancer cells. Biochem. Biophys. Res. Commun. 493, 934–939. 10.1016/j.bbrc.2017.09.117 28951214

[B34] Xu Y. Mizuno T. Sridharan A. Du Y. Guo M. Tang J. (2016). Single-cell RNA sequencing identifies diverse roles of epithelial cells in idiopathic pulmonary fibrosis. JCI Insight 1, e90558 10.1172/jci.insight.90558 27942595 PMC5135277

[B35] Yamada A. Aki T. Unuma K. Funakoshi T. Uemura K. (2015). Paraquat induces epithelial-mesenchymal transition-like cellular response resulting in fibrogenesis and the prevention of apoptosis in human pulmonary epithelial cells. PLoS One 10, e0120192 10.1371/journal.pone.0120192 25799450 PMC4370722

[B36] Yamada M. Kuwano K. Maeyama T. Hamada N. Yoshimi M. Nakanishi Y. (2008). Dual-immunohistochemistry provides little evidence for epithelial-mesenchymal transition in pulmonary fibrosis. Histochem. Cell Biol. 129, 453–462. 10.1007/s00418-008-0388-9 18236067

[B37] Zhan T. Rindtorff N. Boutros M. (2017). Wnt signaling in cancer. Oncogene 36, 1461–1473. 10.1038/onc.2016.304 27617575 PMC5357762

[B38] Zhong Y. Lee K. Deng Y. Ma Y. Chen Y. Li X. (2019). Arctigenin attenuates diabetic kidney disease through the activation of PP2A in podocytes. Nat. Commun. 10, 4523 10.1038/s41467-019-12433-w 31586053 PMC6778111

[B39] Zhu Z. Yan J. Jiang W. Yao X. G. Chen J. Chen L. (2013). Arctigenin effectively ameliorates memory impairment in Alzheimer’s disease model mice targeting both β-amyloid production and clearance. J. Neurosci. 33, 13138–13149. 10.1523/JNEUROSCI.4790-12.2013 23926267 PMC6619735

[B40] Zyoud S. H. (2018). Investigating global trends in paraquat intoxication research from 1962 to 2015 using bibliometric analysis. Am. J. Ind. Med. 61, 462–470. 10.1002/ajim.22835 29537078

