# Supplementary figures and images for: Arctigenin Suppressed Epithelial-Mesenchymal Transition Through Wnt3a/β-Catenin Pathway in PQ-Induced Pulmonary Fibrosis
Source: Front Pharmacol. 2020 Dec 16;11:584098. doi: 10.3389/fphar.2020.584098 (PMC7772408; doi:10.3389/fphar.2020.584098)

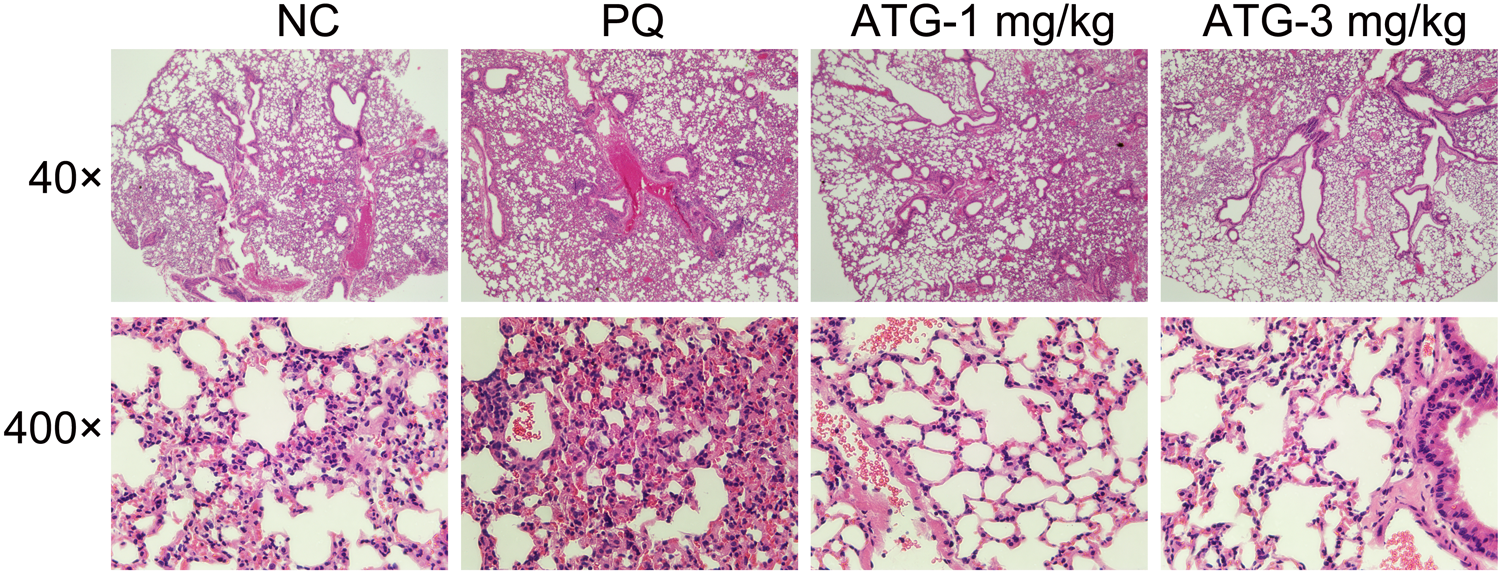

Supplement: Supplementary file 1 [file Image1.TIF]
